# Draft Genome Sequence and Complete Plasmid Sequence of *Acinetobacter lwoffii* F78, an Isolate with Strong Allergy-Protective Properties

**DOI:** 10.1128/genomeA.00685-16

**Published:** 2016-07-21

**Authors:** Moritz Fritzenwanker, Torsten Hain, Dörthe A. Kesper, Hani Harb, Harald Renz, Eugen Domann

**Affiliations:** a Institute for Medical Microbiology, German Centre for Infection Research (DZIF Partner Site Giessen-Marburg-Langen), Justus-Liebig-University Giessen, Giessen, Germany; b Institute of Laboratory Medicine, Pathobiochemistry and Molecular Diagnostics, Philipps University, Marburg, Germany

## Abstract

The hygiene hypothesis states that the tremendous increase in atopic diseases correlates significantly with less contact to microbes in childhood. Here, we report the draft genome sequence of *Acinetobacter lwoffii* F78, a rural cowshed isolate with strong allergy-protective properties that contains an 8,579-bp plasmid.

## GENOME ANNOUNCEMENT

Susceptibility to asthma and allergic diseases is complex and involves genetic variants and environmental exposure (such as exposure to bacteria, viruses, and pets), alteration of our microbiome, and the large-scale manipulation of the environment over the past century. These findings led to the hygiene hypothesis, which states that the tremendous increase in atopic diseases correlates with less contact to microbes and fewer infections in childhood. A number of epidemiological studies have shown that children who grow up in a farming environment will develop less atopic disorders later in life (1–5).

The analysis of cowshed microflora from a farming environment in Bavaria (Germany) revealed one abundant bacterium, *Acinetobacter lwoffii* isolate F78, which was able to reduce allergic reactions in mice, activate mammalian cells *in vitro*, and induce a Th1-polarizing program in dendritic cells. The allergy-protective properties of this isolate were found to be imparted by its lipopolysaccharide (6–8).

*Acinetobacter* spp. are common in nature and widely distributed in the hospital environment and are capable of causing nosocomial infections. The genus is able to survive on moist and dry surfaces and is present in foodstuffs and on healthy human skin. In general, *Acinetobacter* spp. are considered to be nonpathogenic to healthy individuals but may cause infections in debilitated and immunocompromised people. *A. baumannii* is the most frequently isolated species from humans, whereas *A. lwoffii* belongs to the predominant species found in food. The microorganism survives desiccation for prolonged periods and grows at a wide range of temperatures (9–11).

DNA sequencing libraries were prepared using the Nextera XT kit (Illumina, USA) according to the manufacturer’s instructions. Individually tagged libraries were sequenced as a part of a flow cell as 2 × 300-bp paired-end reads using the Illumina MiSeq platform. A total of 12,447,167,642 sequences were produced, and the sequences from each isolate were separately assembled using CLC Genomics Workbench version 7.0.4. The location of open reading frames and the annotation of genes were done using RAST (http://rast.nmpdr.org), and a genetic map of the resulting contigs was generated with Mauve (12, 13). The 8,579-bp plasmid was designated pAlw-F78.

### Nucleotide sequence accession numbers.

This whole-genome shotgun project has been deposited at DDBJ/EMBL/GenBank under the accession numbers FLLN01000001 to FLLN01000012. The versions described in this paper are the first versions.
